# Vitamin D Intake and the Risk of Colorectal Cancer: An Updated Meta-Analysis and Systematic Review of Case-Control and Prospective Cohort Studies

**DOI:** 10.3390/cancers13112814

**Published:** 2021-06-04

**Authors:** Hatim Boughanem, Silvia Canudas, Pablo Hernandez-Alonso, Nerea Becerra-Tomás, Nancy Babio, Jordi Salas-Salvadó, Manuel Macias-Gonzalez

**Affiliations:** 1 Instituto de Investigación Biomédica de Málaga (IBIMA), Unidad de Gestión Clínica de Endocrinología y Nutrición del Hospital Virgen de la Victoria, 29010 Málaga, Spain; hatim.boughanem@ibima.eu (H.B.); manuel.macias@ibima.eu (M.M.-G.); 2 Universitat Rovira i Virgili, Departament de Bioquímica i Biotecnologia, Unitat de Nutrició Humana, Hospital Universitari San Joan de Reus, 43201 Reus, Spain; silvia.canudas@gmail.com (S.C.); nancy.babio@urv.cat (N.B.); jordi.salas@urv.cat (J.S.-S.); 3 Institut d’Investigació Sanitària Pere Virgili (IISPV), 43204 Reus, Spain; 4 Instituto de Salud Carlos III (ISCIII), Consorcio CIBER, M.P. Fisiopatología de la Obesidad y Nutrición (CIBERObn), 28220 Madrid, Spain; 5 Department of Nutrition, Food Sciences and Gastronomy, Food Torribera Campus, School of Pharmacy and Food Sciences, University of Barcelona, Santa Coloma de Gramenet, 08921 Barcelona, Spain; 6 Biochemical and Biotechnology Department, Faculty of Medicine and Health Sciences Human Nutrition Unit, Rovira and Virgili University, C/Sant Llorenç, 21, 43201 Reus, Spain; 7 Open Evidence Research Group, Universitat Oberta de Catalunya, 08018 Barcelona, Spain; 8 Department of Preventive Medicine and Public Health, School of Medicine, University of Valencia, 46010 Valencia, Spain; 9 MRC Centre for Environment and Health, Department of Epidemiology & Biostatistics, School of Public Health, Faculty of Medicine, Imperial College London, St Mary’s Campus, Norfolk Place, London W2 1PG, UK

**Keywords:** vitamin D intake, meta-analysis, systematic review, colorectal cancer, incidence, case-control, prospective

## Abstract

**Simple Summary:**

Colorectal cancer (CRC) is the third most diagnosed cancer in men and the second in women worldwide, being the second most deadly cancer worldwide. The evidence coming from experimental studies suggest a protective effect of vitamin D intake on the risk of CRC. Different studies have shown that vitamin D may play a chemopreventive role in colorectal adenoma incidence, malignant transformation and progression. Our objective was to conduct an updated systematic review and meta-analysis of both case-control and prospective cohort studies on vitamin D intake and CRC. This manuscript provides a complete and updated state-of-the-art about vitamin D intake and CRC risk.

**Abstract:**

Obesity, a sedentary lifestyle, high red meat consumption and alcohol, and tobacco are considered the driving factors behind colorectal cancer (CRC) worldwide. Both diet and lifestyle are recognized to play an important role in the prevention of CRC. Forty years later, the vitamin D–cancer hypothesis is considered consistent. However, the relationship between low vitamin D intake and CRC is still controversial. The aim of this meta-analysis is to determine the associations between Vitamin D intake and CRC. MEDLINE-PubMed and Cochrane databases were searched up to May 2020 for studies evaluating the association between vitamin D intake (from foods and supplements) and CRC. Two reviewers, working independently, screened all titles and abstracts to identify the studies that met the inclusion criteria (case-control or prospective cohort (PC) studies published in English). Data were pooled by the generic inverse variance method using a random or fixed effect model. Heterogeneity was identified using the Cochran Q-test and quantified by the I^2^ statistic. A total of 31 original studies were included for the quantitative meta-analysis, comprising a total 47.540 cases and 70.567 controls in case-control studies, and a total of 14.676 CRC-incident cases (out of 808.130 subjects in PC studies) from 17 countries. A significant 25% lower risk was reported comparing the highest vs. the lowest dietary vitamin D consumption and CRC risk (odds ratio (95% confidence interval): 0.75 (0.67; 0.85)) in case-control studies, whereas a non-significant association was reported in case of prospective studies (hazard ratio (95% confidence interval): 0.94 (0.79; 1.11). The present meta-analysis demonstrates that high dietary vitamin D is associated to CRC prevention. However, larger and high-quality prospective studies and clinical trials are warranted to confirm this association.

## 1. Introduction

Colorectal cancer (CRC) is the third most commonly diagnosed cancer in men and the second in women, being the second most deadly cancer worldwide, with about 881,000 deaths estimated for 2018 [1]. Environmental and genetic factors play a major role in the pathogenesis of CRC. Risk factors for CRC include aging, family history of CRC, medical history of benign adenomatous polyps and inflammatory bowel diseases, obesity, diabetes, lack of physical exercise, and diet [2]. The World Cancer Research Fund (WCRF) and the American Institute for Cancer Research (AICR) has recognized with strong evidence a decrease in the risk of CRC when consuming wholegrains, foods containing dietary fiber and dairy products. However, the consumption of high amounts of red and/or processed meat, and alcoholic drinks have been associated to an increased risk of CRC. The same institutions have categorized the consumption of fish and low intake of non-starchy vegetables and fruits with limited suggestive evidence of association with CRC risk. While it is not clear by which mechanisms this diet modulates cancer risk, there is substantial metabolic and experimental evidence to implicate fiber and antioxidant micronutrients.

Since Garland et al. [3] in 1980, proposed vitamin D for colon cancer (CC) prevention, experimental studies with vitamin D have demonstrated evidence for its antitumor effect, especially in CRC [4]. In vitro and in vivo experiments have suggested a special role of vitamin D response that may explain the potentially protective effects of vitamin D against CRC [5,6].

Regarding dietary vitamin D intake, the WCRF/AICR report in 2017 concluded that the evidence of a protective effect of vitamin D on the risk of CRC was limited suggestive [7]). A recent meta-analysis of 166 prospective studies published in 2019 showed that: (1) Colorectal adenoma incidence was inversely correlated with the circulating 25-hydroxyvitamin D (25(OH)D) level and vitamin D intake; (2) the CRC incidence was decreased by circulating 25(OH)D and vitamin D intake; (3) high-level circulating 25(OH)D triggered better CRC-specific survival; and (4) circulating 25(OH)D decreased colorectal adenoma and CRC risk in populations with higher calcium intake [8]. These results suggest that vitamin D may play a chemopreventive role in colorectal adenoma incidence, malignant transformation, and progression to CRC.

Because of this suggestive evidence coming from epidemiologic studies between dietary vitamin intake and CRC, our objective was to conduct an updated systematic review and meta-analysis of case-control and prospective cohort (PC) studies on CRC and vitamin D intake. This article provides a complete and updated state-of-the-art about vitamin D intake and CRC risk, while considers putative differences coming from sex, sources of vitamin D (i.e., dietary, supplemental, and total) and was conducted separately in case-control and PC studies.

## 2. Materials and Methods

### 2.1. Search Strategy and Study Selection

For the present systematic review and meta-analysis, we followed the methodological guidelines of the Cochrane Handbook for Systematic Reviews of interventions [9] and the results were reported according to Meta-analysis of Observational Studies in Epidemiology (MOOSE) guidelines [10]. The present study and the corresponding search protocol have been registered in the PROSPERO registry (http://www.crd.york.ac.uk/) (accessed on 7 September 2020; ROSPERO) as CRD42020155587.

We conducted a comprehensive electronic systematic search in two databases (Medline through PUBMED and Cochrane Library) until 5th May 2020 combining different MeSH terms and key words. PubMed search was conducted using R packages “pubmed.mineR” and “RISmed”. Table S1 depicts detailed search strategy. Additionally, a manual review of the reference list from the retrieved articles was conducted to ensure that all relevant studies conducted in the field were identified.

In the first step, duplicate studies from the identified articles through the search strategy were discarded. In a second step, two independent reviewers (HB and SC) performed an initial screening of the titles and abstracts of the retrieved papers against the eligibility criteria. For that purpose, the Abstrackr (http://abstrackr.cebm.brown.edu/) (accessed on 7 September 2020) online screening program was used. Their selection was supervised by PH-A and NB-T.

Eligible studies were those with case-control or with PC design with at least 1-year of follow-up, conducted on adults (≥18 years old), and reporting the risk estimates as odd ratios (OR) or hazard ratios (HR) and their corresponding 95% confidence interval (CI) for the association between vitamin D intake—including dietary, supplements, and/or total intake and the risk of CRC, CC, and/or rectal cancer (RC). We did not consider for inclusion published abstracts or proceedings.

### 2.2. Data Extraction

In order to verify that the articles that passed the previous process met the eligibility criteria, two independent researchers (HB and SC) reviewed the full text. In addition, they also extracted relevant information for the systematic review and meta-analysis of each of the studies using a standardized spreadsheet proforma. Collected data included authors, journal and year of publication, title of the article, study name, participant characteristics, sample size, follow-up (only for cohort studies), type of exposure, dietary assessment method, type of outcome and assessment method, number of cases, statistical analyses, and multivariable-adjusted risk estimates (OR or HR, and 95%CI) for the association of interest. Disagreements between researchers were solved by consensus or consulting a third researcher (PH-A).

### 2.3. Quality Assessment of the Included Studies

Two different tools were used to assess the quality of the included studies. For case-control studies, we used The Study Quality Assessment of Case-Control Studies from the National Heart, Lung, and Blood Institute [11]. It consists of 12 questions that help the researcher to rate the studies as good, fair, or poor based on details that are reported in the studies. Poor quality is translated to high risk of bias, and good quality as low risk of bias.

For prospective cohort studies we used the Newcastle-Ottawa Scale [12]. This is a rating scale ranging from 0 to 9 points that are given to the studies based on three domains. A maximum of 4, 3, and 2 points are given after evaluating the population selection, outcome assessment, and comparability domains, respectively. Studies with a total punctuation of at least 7 points were considered as high quality. Any disagreement between the researchers (HB and SC) was solved by consensus or consulting a third researcher (PH-A).

### 2.4. Vitamin D Intake and Colorectal Cancer Outcomes

In this meta-analysis we have considered as exposure the intake of vitamin D from foods (i.e., dietary vitamin D), from supplements (i.e., supplemented vitamin D), and the composite of the previous two (i.e., total vitamin D intake). Moreover, we considered the outcomes: CRC and its subsites (i.e., CC and RC). However, the main exposure and outcome were dietary vitamin D and CRC respectively, in the overall population (i.e., all subjects). Importantly, results from the same study were included in the meta-analysis when data were reported by non-overlapping subjects (i.e., men and women to create all subjects). However, independent meta-analyses were performed for studies comprising only men, only women, or including both as it has been shown that for CRC and its subsites the risk is sex-dependent [13].

### 2.5. Statistical Analyses

We conducted all the analyses using R version 3.6.3 software including packages “meta” (v. 4.11) and “dmetar” (v. 0.0.9). The natural log-transformed ORs, HRs, and 95% CI comparing highest versus lowest categories of vitamin D intake were pooled using the generic inverse variance method with fixed-effects model (when less than 5 study comparisons were available) or random-effects model (when at least 5 or more study comparisons were available). The results were reported back in the original scale. Studies using continuous risk per dose were excluded from the analyses, but we described their results within the text. We conducted separated meta-analyses for case-control and prospective cohort studies.

For all meta-analyses, the Cochran Q statistic was used to estimate inter-study heterogeneity and it was quantified by the I^2^ statistic. We considered substantial heterogeneity when I^2^ was ≥ 50% and P_heterogeneity_ < 0.10. We additionally reported the tau^2^ as the estimate of the between-study variance in random-effects meta-analyses.

Sensitivity analyses were conducted when more than four study comparisons (from independent studies) were available in the analyses by the removal of one study at a time (i.e., leave-one out approach) from the meta-analyses and recalculating the summary risk estimates and heterogeneity values. We considered an influential study that changed the evidence of heterogeneity or the magnitude by more than 20%, the significance and/or direction of the association. Moreover, the detection of outliers (i.e., study’s confidence interval does not overlap with the confidence interval of the pooled effect) was also performed. Finally, a graphic display of heterogeneity (GOSH) plot was performed to test all the possible study combinations within a meta-analysis (2^n^ − 1 individual analyses, where “n” is the number of studies) and then plot the pooled effect size on the *x*-axis and the between-study heterogeneity at the *y*-axis.

Publication bias—by means of a funnel plot to visually assess small study effects—is only possible to be tested when ten or more study comparisons are included in a meta-analysis [14]. Therefore, we only performed them in the meta-analysis of case-control studies assessing the risk of CRC by dietary vitamin D intake, in all the subjects.

## 3. Results

### 3.1. Study Characteristics

**Figure 1:**
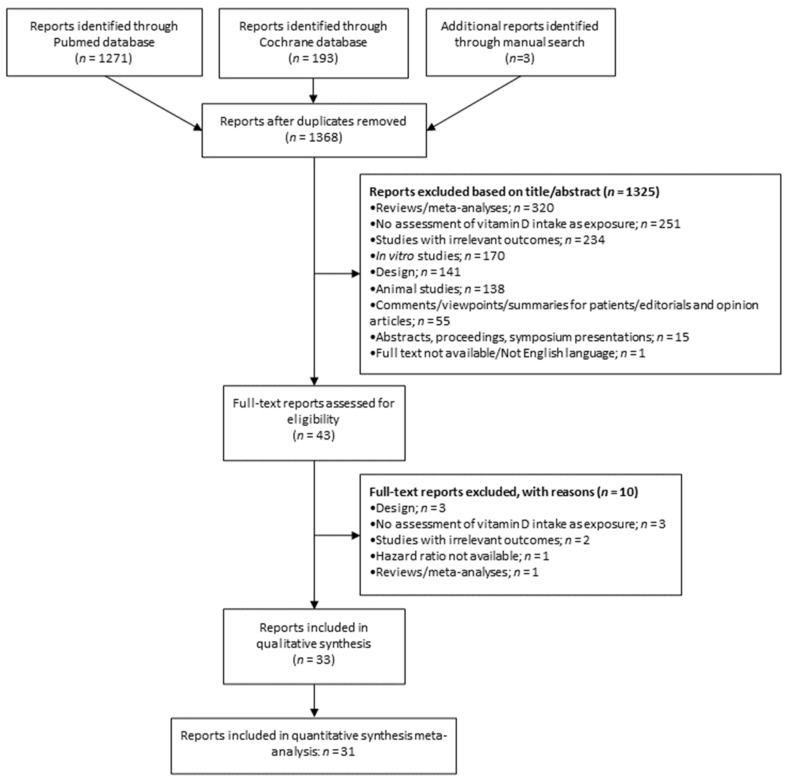
Flowchart of the studies included in this meta-analysis.

A primary search of MEDLINE-PubMed and Cochrane databases, together with manual search, retrieved a total of 1320 articles (Figure 1) after duplicates were removed. About 96.8% (*n* = 1278) were excluded based on their title and abstract information according to the eligibility criteria. Therefore, 42 articles were collected as full texts and were further assessed for inclusion. A total of 33 articles were included in the qualitative synthesis, and 31 (which reported risk estimates comparing extreme categories) were included in quantitative synthesis meta-analysis (Figure 1).

Information regarding each study is depicted in Table 1 and Table 2. This meta-analysis included a total of 47.540 cases and 70.567 controls in case-control studies, and a total of 14.676 CRC-incident subjects (out of 808.130 subjects) in PC studies, from 16 countries around the world. The follow-up in the PC studies ranged from 5 to 16 years. Most of the studies assessed vitamin D intake through validated FFQ or using a 24-h dietary recall. Some studies stratified the analysis by sex. Therefore, we considered these results separately in each corresponding meta-analysis.

Regarding the quality of the studies, the vast majority of case-control studies were evaluated as “Good” (81%) and the remaining as “Fair”. All the prospective studies were qualified a mark at least “7/9”, with 75% of them having a score “8/9”. Estimate risks from two case-control studies (Peters et al. 1992 [15] and Vallès et al. 2018 [16]) were only reported on a continuous scale, instead of categories of vitamin D intake (i.e., highest versus lowest). Moreover, partial data from a case-control study (La Vecchia et al. 1997 [17]) and a PC (Martínez et al. 1996 [18]) were also reported as continuous. These data were not meta-analyzed but remarked as appropriate.

**Table 1:** Characteristics of case-control studies included in the systematic review and meta-analysis (vs.).

Study	Country	Study Name	Controls (M/F)	Cancer Type	Cases (M/F)	Vitamin D Intake	Vitamin D Source	Age (Years)	Quality ^a^
Peters et al. 1992 [15]	USA		746	CC	746 (419/327)	Continuous	Dietary	45–69	GOOD
Ferraroni et al. 1994 [19]	Italy		2024 (1189/835)	CRC CC RC	CRC: 1326 (711/615) CC: 828 RC: 398	Q5 vs. Q1	Dietary	20 to 74	FAIR
Olsen et al. 1994 [20]	Denmark		759 (438/321)	CRC	49	T3 vs. T1	Dietary	45 to 74	GOOD
Boutron et al. 1996 [21]	France		309 (159/150)	CRC	171 (109/62)	Q5 vs. Q1	Dietary	30 to 75	GOOD
Pritchard et al. 1996 [22]	Sweden		512 (276/236)	RC CC	RC: 217 (107/110) CC: 352 (189/163)	Qu4 vs. Qu1 ^b^	Dietary	67.7 (9.0)	FAIR
La Vecchia et al. 1997 [17]	Italy	Case control study Italy	4154 (2073/2081)	CRC CC ^c^ RC ^c^	CRC 1953 (1125/818) CC: 1225 RC: 728	Q5 vs. Q1	Dietary	23 to 74	GOOD
Marcus et al. 1998 [23]	USA		678 F	CC RC	CC: 348 F RC: 164 F	Q5 vs. Q1	Dietary Supplemental Total	<75	FAIR
Kampman et al. 2000 [24]	USA		2400 (1114/1286)	CC	1993 (1095/888)	Q5 vs. Q1	Dietary Supplemental (Ever vs. Never)	30 to 79	GOOD
Levi et al. 2000 [25]	Switzerland		491 (211/280)	CRC	223 (142/81)	T3 vs. T1	Dietary	27 to 74	GOOD
Slattery et al. 2004 [26]	USA	KPMCP and the state of Utah	1197 (672//525)	RC	RC: 946 (556/390)	Four categories (highest vs. lowest) ^d^	Dietary	30 to 79	GOOD
Mizoue et al. 2008 [27]	Japan	Fukuoka Colorectal Cancer Study	861 (327/534)	CRC CC RC	CRC: 836 (502/334) CC: 476 RC: 354	Q5 vs. Q1	Dietary	20 to 74	GOOD
Theodoratou et al. 2008 [28]	UK	SOCCS	2793 (1591/1202)	CRC	2070 (1185/885)	Q5 vs. Q1	Dietary Total	16 to 79	GOOD
Lipworth et al. 2009 [29]	Italy		4154 (2073/2081)	CC RC	CC: 1225 (688/537) RC: 728 (437/291)	D10 vs. D1	Dietary	20–74	GOOD
Jenab et al. 2010 [30]	Europe ^e^	EPIC		CRC CC RC	CRC: 1248 (620/628) CC: 785 (369/416) RC: 463 (251/212)	Q5 vs. Q1	Dietary	30 to 77	GOOD
Key et al. 2011 [31]	UK	UK Dietary Cohort Consortium	1951 (980/971)	CRC	565 (266/299)	Four categories (highest vs. lowest)	Dietary	62.2 (9.2)	GOOD
Sun et al. 2011 [32]	Canada	NL and ON cohorts	NL: 488 ON: 1830	CRC	NL: 651 ON: 1272	Q5 vs. Q1	Supplemental	20 to 74	GOOD
Banqué et al. 2012 [33]	Spain		490 (312/178)	CRC	245 (156/89)	T3 vs. T1	Dietary	30 to 80	GOOD
Sun et al. 2012 [34]	Canada	NL and ON cohorts	2481 (1357/1124)	CRC	1760 (935/825)	Q5 vs. Q1	Dietary Total	20 to 74	GOOD
Vallès et al. 2018 [16]	Spain	MCC-Spain	3950 (2018/1932)	CRC	2140 (1365/445)	Continuous	Dietary	20 to 85	FAIR
Hosseinzadeh et al. 2019 [35]	Iran		201 (108/93)	RC	162 (94/68)	Adequate vs. low intake	Dietary	40 to 80	GOOD
Zhang et al. 2020 [36]	China		2389	CRC CC RC	CRC: 2380 (1356/1924) CC: 1476 RC: 828	Qu4 vs. Qu1 ^b^	Dietary	30 to 75	GOOD

^a^ assessed by the Study Quality Assessment Tools from the National Heart, Lung and Blood Institute for case-control studies. ^b^, includes combined results for men and women, and separated results. ^c^, continuous. ^d^, includes separated results for men and women. ^e^, includes Denmark, France, Greece, Germany, Italy, the Netherlands, Norway, Spain, Sweden, and the United Kingdom. Results from Sun et al. 2011 regarding dietary and total vitamin D are included as the combination of both NL and ON cohorts in Sun et al. 2012. Funding source for all the studies is agency. Abbreviations: CC, colon cancer; CRC, colorectal cancer; D, decile; EPIC, European Prospective Investigation Into Cancer and Nutrition; F, females; FFQ, food frequency questionnaire (self-administered); KPMCP, Kaiser Permanente Medical Care Program of Northern California; M, males; MCC, multicenter case-control; NA, not applicable; NL, Newfoundland and Labrador subjects; ON, Ontario subjects; Qu, quartile; Q, quintile; RC, rectal cancer; SOCCS, Study of Colorectal Cancer in Scotland; T, tertile; wk, week.

**Table 2:** Characteristics of prospective studies included in the systematic review and meta-analysis.

Study	Country	Study Name	Total Population (M/F)	Cancer Type	Incident Cases (M/F)	Vitamin D Intake	Vitamin D Source	Age (Years)	Follow-UP ^a^	Quality ^b^
Bostick et al. 1993 [37]	USA	The Iowa Women’s Health Study cohort	35216 F	CC	212	Q5 vs. Q1	Dietary Supplemental (T3 vs. T1) Total	55 to 69	5 years	8/9
Kearney et al. 1996 [38]	USA	The Health Professionals Follow-up Study	47935 M	CC	203	Q5 vs. Q1	Dietary Supplemental Total	40 to 75	6 years	8/9
Martínez et al. 1996 [18]	USA	Nurses’ Health Study	89448 F	CRC CC ^c^ RC ^c^	CRC: 501 CC: 396 RC: 105	Q5 vs. Q1	Dietary Total	30 to 55	12 years	8/9
Zheng et al. 1998 [39]	USA		34702 F	RC	144	T3 vs. T1	Dietary	55 to 69	9 years	8/9
Järvinen et al. 2001 [40]	Finland		9959	CRC CC RC	CRC: 72 CC: 38 (15/23) RC: 34 (21/13)	Qu4 vs. Qu1	Dietary	>15	24 years	8/9
Terry et al. 2002 [41]	Sweden	Swedish Mammography Screening Cohort	61463 F	CRC CC RC	CRC: 517 CC: 371 RC: 191	Q5 vs. Q1	Dietary	40 to 74	11.3 years	9/9
McCullough et al. 2003 [42]	USA	Cancer Prevention Study II Nutrition Cohort	127749 (60,866/66,883)	CRC	692 (421/271)	Q5 vs. Q1 ^d^	Dietary	50 to 74	5 years	8/9
Keese et al. 2005 [43]	France	E3N-EPIC	67,312 F	CRC	172	Qu4 vs. Qu1	Dietary	40 to 65	6.9 years	8/9
Lin et al. 2005 [44]	USA	US Women’s Health Study	36,976 F	CRC	223	Q5 vs. Q1	Dietary Supplemental (yes vs. no) Total	>45	10 years	8/9
Park et al. 2007 [45]	USA	Multiethnic Cohort Study (Hawaii and Los Angeles, California	191011 (85903/105108)	CRC	2110 (1138/972)	Q5 vs. Q1 ^d^	Dietary Supplemental Total	45 to 75	7.3 years	7/9
Ishihara et al. 2008 [46]	Japan	The Japan Public Health Center-based Prospective Study	71138 (35193/35945)	CRC	761 (464/297)	Q5 vs. Q1 ^e^	Dietary	45 to 74	7.8 years	8/9
Um et al. 2018 [44]	USA	Iowa Women’s Health Study	35,221 F	CRC	1731	Yes/No	Supplemental	55 to 69	16 years	7/9

^a^, mean, median or range ^b^, assessed by the The Newcastle-Ottawa Scale. ^c^, continuous ^d^, includes combined results for men and women, and separated results. ^e^, includes separated results for men and women. Abbreviations: CC, colon cancer; CRC, colorectal cancer; F, females; FFQ, food frequency questionnaire (self-administered); M, males; NA, not applicable; Qu, quartile; Q, quintile; RC, rectal cancer; T, tertile; wk, week.

### 3.2. Meta-Analyses of Case-Control Studies

#### 3.2.1. Colorectal Cancer

**Figure 2:**
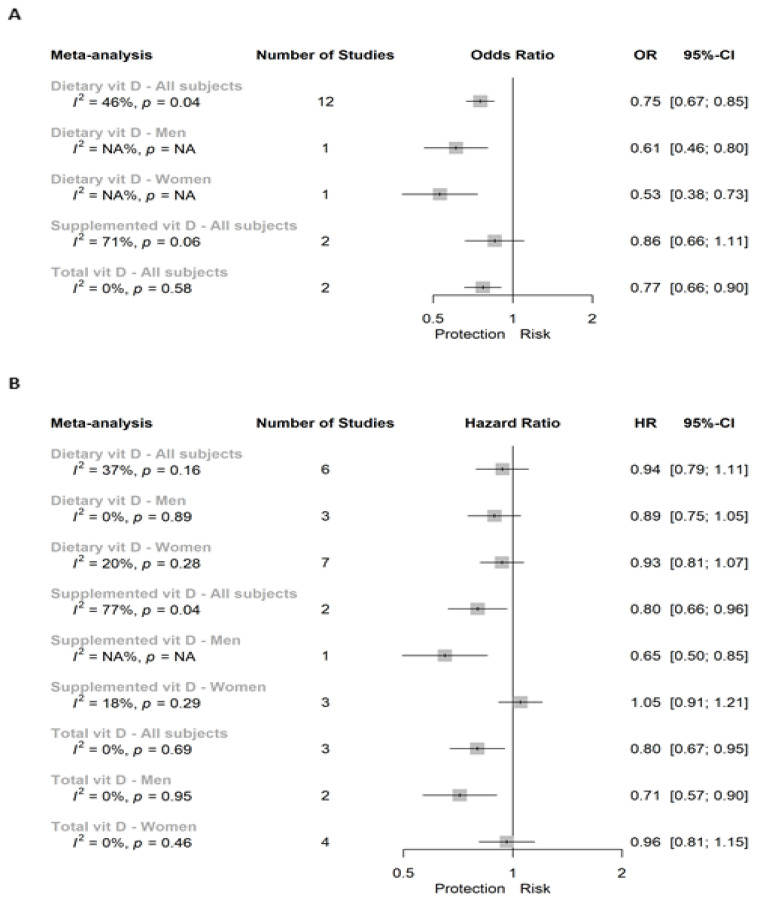
Super plot of (**A**) case-control and (**B**) prospective cohort studies assessing the association between vitamin D intake (highest versus lowest categories) and the risk of colorectal cancer.

**Figure 3:**
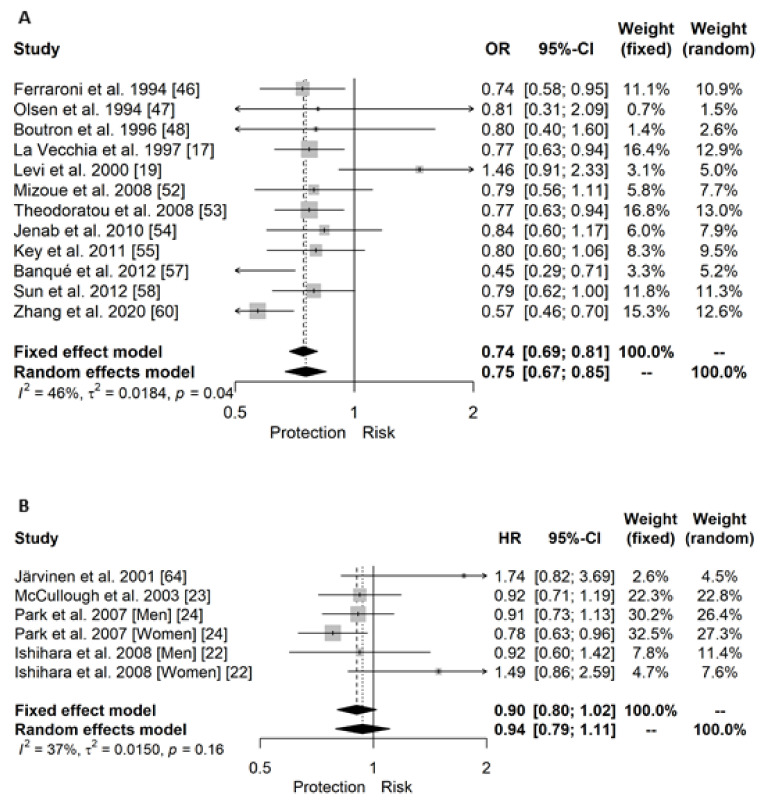
Forest plot for the association between dietary vitamin D intake (highest versus lowest categories) and risk of colorectal cancer including all subjects for (**A**) case-control and (**B**) prospective studies.

A total of three independent meta-analyses were performed for case-control studies assessing the risk of CRC by dietary, supplemental, or total vitamin D intake when considering all the subjects (Figure 2A). Specifically, a significant 25% lower risk was reported between dietary vitamin D consumption and CRC risk (OR (95% CI): 0.75 (0.67; 0.85)). Figure 3 shows the forest plot for that meta-analysis. One study reported this association separated by sex and significant inverse association was showed in both sexes (Figure 2A). This significant inverse association was also seen in case of total vitamin D (0.77 (0.66; 0.90); (forest plot in Figure S1B), whereas it was not significant in case of supplemented vitamin D (0.86 (0.66; 1.11), forest plot in Figure S1A). In a continuous scale, results from Vallès et al. 2018 [16] showed a non-significant association (0.96 (0.89; 1.03)) between dietary vitamin D and CRC risk in a population twice represented of men versus women.

#### 3.2.2. Colon Cancer

**Figure 4:**
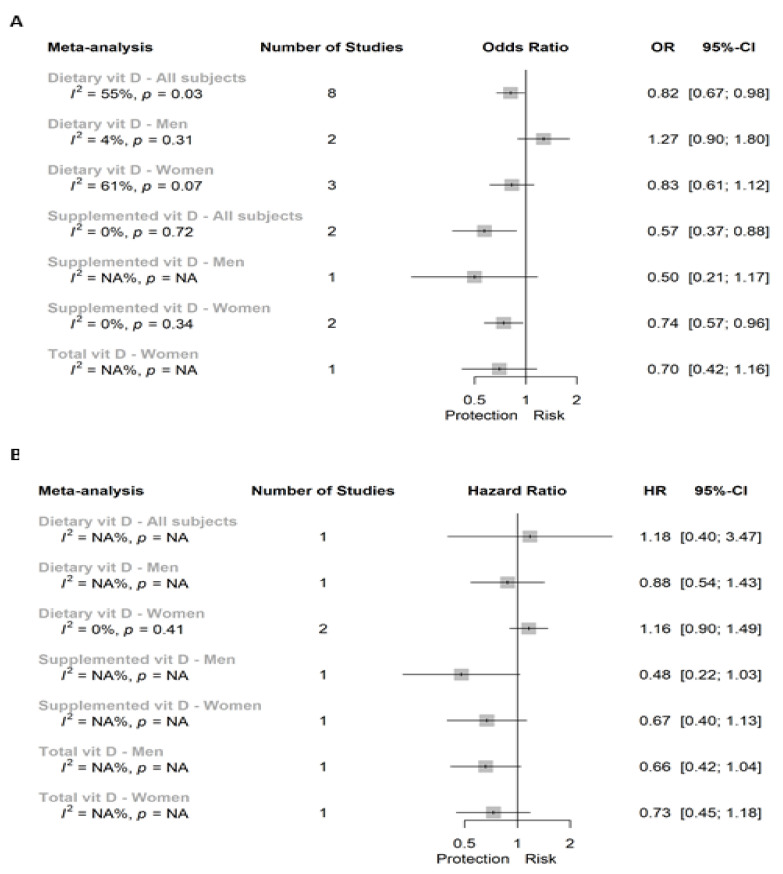
Super plot of (**A**) case-control and (**B**) prospective cohort studies assessing the association between vitamin D intake (highest versus lowest categories) and the risk of colon cancer.

In Figure 4A we show the results of the case-control studies assessing the association between vitamin D intake and CC. We found a significant 18% lower risk of CC in those individuals in the highest versus the lower category of dietary vitamin D intake when considering all subjects (OR (95% CI): 0.82 (0.67; 0.98)), but not when we separately analyzed the associations in men or women alone (Figure S2A–C, respectively).

Associations between vitamin D supplementation and CC differed by sex, toward a significant inverse association for all subjects (0.57 (0.37; 0.88)) and studies conducted in women (0.74 (0.57; 0.96); Figure S2D,E), but not in case of the unique study in men (Figure 4A). Finally, total vitamin D was only assessed in one study in women, and showed a non-significant association in case-control studies (Figure 4A).

In a continuous scale, Peters et al. 1992 [15] reported the associations between CC and dietary vitamin D in all subjects, men only, and women only, showing non-significant positive associations in all of them (1.08 (0.97; 1.2); 1.1 (0.95; 1.26); and 1.08 (0.9; 1.28), respectively). However, La Vecchia et al. 1997 [17] reported a significant inverse association for all subjects (0.81 (0.70; 0.90)).

#### 3.2.3. Rectal Cancer

**Figure 5:**
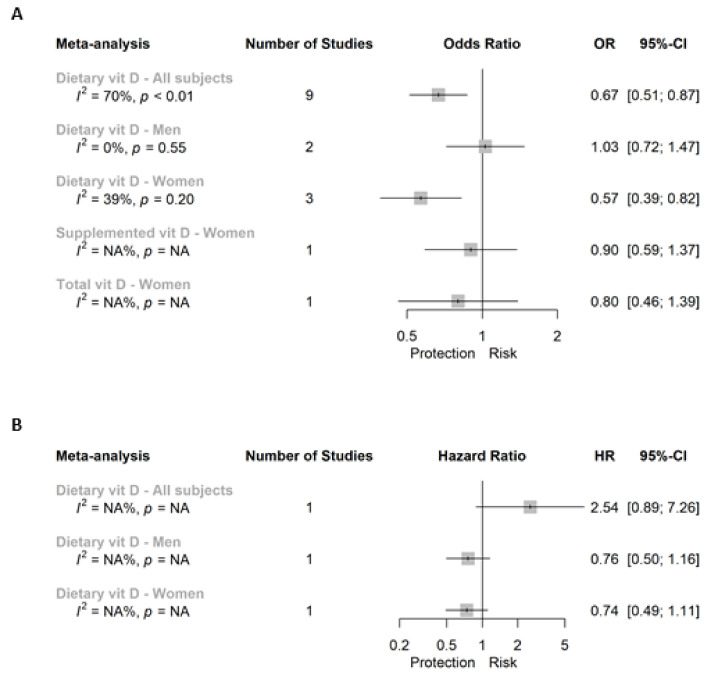
Super plot of (**A**) case-control and (**B**) prospective cohort studies assessing the association between vitamin D intake (highest versus lowest categories) and the risk of rectal cancer.

Results specific for RC are summarized in Figure 5. A total of three meta-analyses reported the association between dietary vitamin D intake and RC risk. We found a significant and inverse association when considering all the subjects (0.67 (0.51; 0.87)) or women alone (0.57 (0.39; 0.82); Figure S3A,C, respectively), whereas we reported a non-significant association in men alone (1.03 (0.72; 1.47); Figure S3B). Specific associations between both supplemental and total vitamin D and RC in women reported non-significant results (Figure 5A).

In a continuous scale, La Vecchia et al. 1997 [17] reported a non-significant association between dietary vitamin D intake and RC in all subjects (1.03 (0.9; 1.2)).

### 3.3. Meta-Analyses of Prospective Cohort Studies

#### 3.3.1. Colorectal Cancer

Figure 2B summarized eight meta-analyses and one independent analysis for the association between dietary intake, supplemental and total vitamin D with CRC incidence in all subjects, and men or women separately. The main outcome referred to dietary vitamin D intake in all subjects, and we did not find a significant association (0.94 (0.79; 1.11); Figure 3B). Moreover, we neither reported a significant association between dietary vitamin D and CRC in men nor in women alone when comparing extreme categories of dietary vitamin D intake (Figure S1C,D, respectively). In the case of supplemental vitamin D, we reported a significant inverse association with CRC incidence in all subjects (0.80 (0.66; 0.96); Figure S1E) and the unique study reporting associations in men (0.65 (0.50; 0.85)), whereas we showed a non-significant association for women (Figure S1F). Finally, this inverse association was also observed when evaluating total vitamin D, toward a 20% and 29% protection in case of all subjects (0.80 (0.67; 0.95)) and men (0.71 (0.57; 0.90)), respectively (Figure S1G,H). However, no significant association was reported in the meta-analysis conducted in women (0.96 (0.81; 1.15); Figure S1I).

#### 3.3.2. Colon Cancer

Figure 4B shows the super plot of six individual analyses and one meta-analysis for the prospective association between vitamin D intake and CC incidence.

The only study conducted assessing the association between dietary vitamin D and CC in all subjects did not show a significant relationship (1.18 (0.40; 3.47)). This non-significant association was also showed in men and women analyzed separately (Figure S2F). The analyses assessing the association between either supplemented or total vitamin D in men or women analyzed separately did not show significant results.

In a continuous scale, Martínez et al. 1996 reported [18], in women only, a non-significant inverse association for both dietary and total vitamin D intake with CC risk (0.96 (0.72; 1.28) and 0.81 (0.63; 1.05), respectively).

#### 3.3.3. Rectal Cancer

Only dietary vitamin D intake and the risk of RC has been evaluated in all subjects, and men or women only. However, in all of them non-significant associations were reported when comparing extreme categories of intake.

In a continuous scale, Martínez et al. 1996 reported [18], in women only, a significant association between dietary vitamin D intake and CC risk (0.45 (0.25; 0.83)), and a non-significant association when total vitamin D was evaluated (1.16 (0.73; 1.82)).

### 3.4. Meta-Analyses Heterogeneity and Publication Bias

#### 3.4.1. Heterogeneity

We did not find substantial heterogeneity in the meta-analyses of case-control studies assessing the association between dietary vitamin D and CRC risk in all subjects (I^2^ = 46%, *p* = 0.04; Figure 2A) nor in the meta-analysis of PC studies (I^2^ = 37%, *p* > 0.10; Figure 2B). In the remaining meta-analyses of CRC, we found substantial heterogeneity in case of both case-control and PC studies evaluating the risk of CRC by supplemental vitamin D in all subjects (I^2^ = 71% and 77%, respectively; *p* < 0.10). In which regards, in colon and RC independently, we reported substantial heterogeneity in the meta-analyses of case-control studies assessing the association between dietary vitamin D intake and both colon (I^2^ = 55%, *p* = 0.03) and rectal (I^2^ = 70%, *p* < 0.01) cancers in all subjects (Figure 4A and Figure 5A, respectively). Moreover, we also reported a substantial 61% of heterogeneity (*p* = 0.07) in case-control studies assessing the risk of CC by dietary vitamin D intake in women alone (Figure 4A).

#### 3.4.2. Publication Bias

Publication bias was assessed using the small sample bias method (Borenstein et al. 2011). The best way to visualize whether small studies with small effect sizes are missing is through funnel plots. Figure S5 shows the funnel plot for the meta-analysis of case-control studies assessing the association between dietary vitamin D and colorectal cancer in all the subjects. All studies lied symmetrically around the pooled OR which was confirmed with a non-significant (*p* = 0.762) value for the Egger’s test of the intercept.

#### 3.4.3. Sensitivity Analysis

Sensitivity analysis with one study removed at a time (leave-one out approach) was performed for analyses of more than four studies in order to assess whether the results could have been substantially affected by a single study. A total of five independent meta-analyses were tested (Table S2). Importantly, in which regards the analyses of outliers, we found one outlier in each case-control, dietary vitamin D intake meta-analysis conducted in all subjects: Levi et al. 2000 [25] in case of CRC (result after its removal: 0.73 (0.66; 0.80)); Kampman et al. 2000 [24] [Men] in case of CC (0.82 (0.67; 0.98)); and Lipworth et al. 2009 [29] in case of RC (0.67 (0.51; 0.87));Table S2). No outliers were detected in the prospective cohort meta-analyses.

In case of case-control studies assessing the association between dietary vitamin D and CRC in all subjects, we found that the exclusion of Levi et al., Banqué et al., or Zhang et al. [25,42,46] significantly reduced the heterogeneity (from 46% to 17%, 35% or 23%, respectively), but had a minimum impact on the pooled OR. In case of case-control studies evaluating dietary vitamin D association with CC, the removal of Kampman et al. 2000 [24] [Men] deeply reduced the heterogeneity from 55% to 10%, although still significant, with an approximately 9% reduction in the OR. The omission of any of the studies did not alter the high heterogeneity for the association between dietary vitamin D and RC in all subjects but did modify the pooled OR.

In the meta-analyses of prospective cohort studies assessing the association between dietary vitamin D and CRC in both all subjects and women alone, no study removal modified the non-significant pooled HRs. However, in case of the meta-analysis of all subjects, the removal of Ishihara et al. 2008 [Men], McCullough et al. 2003, or Park et al. 2007 [Men] [45,47,48], changed the non-significant I^2^ from 7% to a significant 50%.

Interestingly, the GOSH plot for the meta-analysis of case-control studies assessing the risk of CRC in all subjects by dietary vitamin D intake showed two patterns related to the inclusion (red color) or exclusion (blue color) of the outlier. In fact, the removal of the outlier, generated a blue cluster toward reduced pooled ORs with null heterogeneity (Figure S4A). Figure S4B reports a high dispersion of pooled OR values and extremes values of heterogeneity (in approximately 60% and 0%) for the meta-analysis of case-control studies assessing the risk of CC by dietary vitamin D intake in all the subjects. This value was reduced to 0% heterogeneity and lower pooled OR values after removing its outlier. A pattern toward protective pooled ORs but with high heterogeneity was shown in the meta-analysis of case-control studies assessing the risk of RC by dietary vitamin D intake in all the subjects (Figure S5C). However, the removal of the outlier reduced the heterogeneity. Figure S4D,E shows the GOSH plots for the meta-analysis of prospective studies assessing the association between dietary vitamin D intake and CRC risk in all subjects or women only, respectively. Both plots showed a dispersion of values around a pooled HR of 1 (i.e., overall estimate of 0)—mirroring its non-significant associations—although with low heterogeneity.

## 4. Discussion

The present study is the first meta-analysis and systematic review, conducted separately in case-control and PC studies, analyzing the association between vitamin D consumption (including dietary and supplemental vitamin D) and the risk of CRC, CC, and RC, where the differences between sexes were also considered. In our meta-analysis, regarding to case-control studies, we found that dietary vitamin D intake was significantly associated with lower risk of CC, RC, and CRC. A significant association in case of supplemental vitamin D intake was only observed in CC, whereas in case of total vitamin D a significant association was observed in case of CRC. In our meta-analysis of prospective studies, total and supplemental vitamin D intake were solely associated with a reduction in CRC risk. The findings of the present study suggest that vitamin D consumption may have a protective role in CRC development, although several considerations must be taken into account in the management of vitamin D intake and the risk of CRC and the evaluation of study designs.

Many systematic reviews and meta-analyses have been performed to assess the relationship between vitamin D intake and CRC risk, although these studies did not distinguish different vitamin D subtypes, such as total, dietary, or supplemental [8,49,50]. Whereas the evidence suggests an inverse relationship between CRC risk and vitamin D intake, the conclusions are not definitive and point out that additional studies are needed. Indeed, most of these systematic reviews and meta-analyses were designed using a pool of prospective and case-control studies, but not separately, which could lead to a misinterpretation of results due to study designs. So far, only one meta-analysis was performed taking into account the PC and case-control studies separately. This meta-analysis, conducted by Huncharek M et al. 2009 [47], concluded that dietary vitamin D was associated with decreased risk of CRC/CC only in case-control studies (pooled RR was 0.93 (95% CI, 0.86 to 0.99)), but not in case of PC studies [47], which was in line with our main finding.

Regarding to meta-analyses with prospective designs conducted in 2011, a previous meta-analysis also observed an inverse association between CRC risk and dietary vitamin D intake. However, and differing from our findings, non-significant results were reported for total vitamin D intake and CRC risk [48], probably because PC studies considering supplemental vitamin D intake were not included in the risk model of CRC. In contrast, this study mirrored our data regarding CC and RC risk and dietary vitamin D intake, as a non-significant association was observed between them [48]. However, another meta-analysis from nine PC studies found that vitamin D intake (total and dietary) was associated with decreased risk of CRC [49]. These findings also differed from our results probably because this study did not include PC studies considering supplemental vitamin D intake.

The first prospective study concerning serum 25(OH)D and the risk of colon cancer was published by Garland et al. (1989), showing a significant protective role of serum 25(OH)D. Since then, many epidemiological studies have been conducted in both human and animal models to explore such associations [50]. However, results coming from these epidemiological studies and additional meta-analyses and systematic reviews have reported either significant inverse associations or non-significant associations in populations worldwide [49,51,52]. This is because these data did not take into account the additional adjustment for cofounding related-variables, such as the design of the studies, sex differences, tumor site and different metabolites of vitamin D (i.e., 25(OH)D, 1,25(OH)_2_D), among others. As for the supplemental vitamin D in all subjects, our results showed that vitamin D was related to 20% reduced CRC risk in PC studies and 43% reduced CC risk in case-control studies. Our results were in line with a meta-analysis which included 17 cohort studies (including case-control and PC) evaluating the association of vitamin D supplements with CRC risk, and in which vitamin D supplement intake was related to a decreased risk of CRC [53]. However, another meta-analysis and systematic review from PC studies did not find significant associations between vitamin D supplements and CRC [54]. Previous studies have also been conducted to find the link of *VDR* polymorphisms in relation to colorectal cancer. Such polymorphisms are frequently named as BsmI, TaqI, ApaI, Tru9I, FokI, and PolyA mononucleotide repeat. Accordingly, the meta-analysis conducted by Touvier et al. (2011) did not observe significant associations between FokI, PolyA, TaqI, Cdx2, and ApaI VDR polymorphisms and CRC risk, although the BsmI polymorphism was associated with a lower CRC risk [48]. Additional studies also related FokI polymorphism with CRC cases [55,56]. However, these findings require a larger population and a multivariate analysis of other established risk factors to confirm this association, since it remains difficult to confirm the association of VDR polymorphisms with CRC.

Taking into consideration the randomized controlled trials (RCTs), it is still unclear whether supplementation with vitamin D reduces the risk of CRC, since data from RCTs are limited and only a few RCTs have failed to demonstrate protection on CRC after vitamin D_3_ supplementation (See Table S3). For instance, in the Women’s Health Initiative (WHI) trial, no effect was found on CRC prevention after 400 IU of vitamin D_3_ supplementation [57]. In addition, daily supplementation with high-dose vitamin D (2000 IU/day) for 5 years (among initially healthy adults in the United States following the VITamin D and OmegA-3 TriaL -VITAL study) did not reduce the incidence of CRC [58]. These inconsistent findings may be due to confounding factors in the selected studies and longer RCT follow-up in order to observe the benefits. In addition, CRC was a secondary outcome of the calculation of the risk of incidence in the RCTs, in which sample size concerning CRC risk was very limited. Additionally, many of these studies used a relatively small amount of vitamin D_3_ and a low follow-up time, up to 7 years. Thus, specific RCTs, which take specific CRC incidence, large sample size and follow-up time, using high, but safety amount of vitamin D are necessary.

The optimal vitamin D intake is a current subject of interest and is essential for clinical outcome and public health for preventing CRC [59,60]. A previous meta-analysis pointed out that the daily intake of dietary vitamin D of 160 IU or more was associated with a reduced risk of CC [61], whereas another quantitative meta-analysis suggested that daily intake of 1000–2000 IU of vitamin D could reduce the incidence of colorectal with minimal risk [62]. The 2011 report on dietary reference intakes for vitamin D pointed to a recommended dietary intake of 600 IU/day for ages 1 to 70 years and 800 IU/day for ages 71 years and older; higher values were not consistently associated with greater benefit [63]. As we observed, although accumulating data suggest an inverse association between higher dietary vitamin D and the risk of CRC, further studies are needed to clarify the recommended dietary and safety amount of vitamin D to reduce the risk of CRC.

Vitamin D belongs to a group of steroids known as secosteroids. In humans, the most common forms of vitamin D are vitamin D_3_ (cholecalciferol) the main dietary form, and vitamin D2 (ergocalciferol) the form mostly used in supplements and fortified foods. Furthermore, vitamin D is synthesized on sun exposure, through ultraviolet B (UV-B) radiation, from 7-dehydrocholesterol in the skin [64]. At the cellular level, CRC cells contain vitamin D receptors (VDR), and express 1-alpha-hydroxylase, and are thus able to convert 25(OH) vitamin D (the metabolite produced in the liver) into the active form of vitamin D calcitriol (1,25(OH)_2_D_3_) [58,63,64]. Activation of these receptors by 1,25(OH)_2_D induces differentiation and inhibits proliferation, invasiveness, angiogenesis, and metastatic potential. Vitamin D exerts potential roles in CRC and its mechanistic effects additionally showed several properties in the prevention of CRC. Cell fate and phenotype are strictly regulated by extracellular signals. The active metabolite of vitamin D, 1,25(OH)_2_D_3_, (calcitriol), inhibits proliferation, induces apoptosis, and promotes epithelial differentiation of human colon cancer cells, through the modulation of key genes in the carcinogenesis signaling pathways [5]. In fact, 1,25(OH)_2_D_3_, is a major regulator of gene expression and exerts its effects by binding to a transcription factor of the nuclear receptor superfamily: the vitamin D receptor (VDR). VDR heterodimerizes with another member of the same family, the retinoid X receptor, and regulates gene expression in a ligand-dependent manner [4]. For instance, vitamin D antagonizes the Wnt/βcatenin signaling pathways. An aberrant Wnt pathway activation is considered a hallmark of CRC. The antiproliferative effect of vitamin D involves multiple pathways, by inhibiting the cyclin-dependent kinase and growth factors, as well as by increasing the activity of TGF-β1 (transforming growth factor ß1) [65,66]. Vitamin D is also well-known as a modulator of differentiation in colon carcinoma. It regulates many genes involved in cell differentiation such as E-cadherin, occludin, and vinculin, as well as it inhibits β-catenin signaling [67,68]. Furthermore, vitamin D acts by a variety of mechanistic effect of action to suppress the carcinogenesis process. However, this effect may depend on the context of action, such as the bioavailability on the specific tissue and the expression of vitamin D receptor (*VDR)*, as well as the in situ concentration of vitamin D and the expression of the enzymes such as cytochrome p450 24A1 (CYP24A1) and 27B1 (CYP27B1), which modulate the active metabolite of vitamin D.

The main strength of the present systematic review and meta-analysis is that it is the first study analyzing the associations between vitamin D intake, supplemental vitamin D, and total vitamin D intake in both case-control and PC studies, and considering the sex of the subjects. In addition, two different databases were used to identify the available case-control and PC about the relationship between vitamin D intake and supplemental vitamin D on the CRC risk, in which a few of the additional articles were identified manually and further added to the analyses. Finally, the research of literature and selected studies, data selection and extraction, was performed by two independent reviewers, which guarantee the lack of missing of related publishing data. However, the study also has several limitations. First, the analysis from case-control studies identified three outliers, which we have removed from the analysis, reducing the heterogeneity of the results. Second, the correct assessment of dietary supplement intake in several studies may be imprecise, being the relationships between vitamin D and colorectal cancer risk partly due to unmeasured or residual confounding leading to biased results. Third, we could not analyze publication bias in a vast majority of the meta-analyses performed since less than ten study comparisons were included in each one. In addition, for some outcomes we could not conduct a meta-analysis because only one study was identified. Therefore, future research is likely to change the observed risk estimates.

## 5. Conclusions

The quantitative results from our systematic review and meta-analyses from case-control and PC studies support the idea that both dietary and supplemental intake of vitamin D are associated with a reduced risk of CRC, which suggests a significant influence of vitamin D on the prevention of CRC. Available data about vitamin D consumption are not definitive on CRC risk and additional longer follow-up studies, adjusted by cofounding variables, such as the nature of study design, exposure of sunlight, type of diet and time exposure, and amount of supplement vitamin D.

## Data Availability

No new data were created or analyzed in this study. Data sharing is not applicable to this article.

## Supplementary Materials

The following are available online at https://www.mdpi.com/article/10.3390/cancers13112814/s1. Table S1. Search strategy. Table S2. Influence analysis using the leave-one out approach for the meta-analyses assessing the association between vitamin D intake (highest versus lowest categories) and the risk of colon, rectal, and colorectal cancer.Table S3. A list of randomized clinical trials of supplemental vitamin D and the risk of colorectal cancer. Figure S1. Forest plot for the association between vitamin D intake (highest versus lowest categories) and risk of colorectal cancer (case-control and prospective studies). Figure S2. Forest plot for the association between vitamin D intake (highest versus lowest categories) and risk of colon cancer (case-control and prospective studies). Figure S3. Forest plot for the association between vitamin D intake (highest versus lowest categories) and risk of rectal cancer (case-control studies). Figure S4. Graphic display of heterogeneity (GOSH) plot analyses for the different meta-analyses. Figure S5. Funnel plot for detecting publication bias in the meta-analysis of case-control studies assessing the association between dietary vitamin D and colorectal cancer in all the subjects.
