# Supplementary material for: Vitamin D Intake and the Risk of Colorectal Cancer: An Updated Meta-Analysis and Systematic Review of Case-Control and Prospective Cohort Studies
Source: Cancers (Basel). 2021 Jun 4;13(11):2814. doi: 10.3390/cancers13112814 (PMC8201292; doi:10.3390/cancers13112814)
Supplement: Supplementary file 1 [file cancers-13-02814-s001.zip › Supplementary materials.pdf]

Supplementary Materials

Table S1: Search strategy

Table S2. Influence analysis using the leave-one out approach for the meta-analyses assessing the association between vitamin D intake (highest versus lowest categories) and the risk of colon, rectal and colorectal cancer.

Figure S1. Forest plot for the association between vitamin D intake (highest versus lowest categories) and risk of colorectal cancer (case-control and prospective studies).

Figure S2. Forest plot for the association between vitamin D intake (highest versus lowest categories) and risk of colon cancer (case-control and prospective studies).

Figure S3. Forest plot for the association between vitamin D intake (highest versus lowest categories) and risk of rectal cancer (case-control studies).

Figure S4. Graphic display of heterogeneity (GOSH) plot analyses for the different meta-analyses.

Figure S5. Funnel plot for detecting publication bias in the meta- analysis of case-control studies assessing the association between dietary vitamin D and colorectal cancer in all the subjects.

Table S1. Search strategy

**PUBMED SEARCH**<br>(using R packages “pubmed.mineR” and “RISmed”)
'("colorectal neoplasms"[MeSh] OR<br>("colorectal"[ALL] AND "neoplasms"[ALL]) OR<br>"colorectal neoplasms"[ALL] OR<br>("colorectal"[ALL] AND "cancer"[ALL]) OR<br>"colorectal cancer"[ALL] OR<br>"colon cancer"[ALL] OR<br>"rectal cancer"[ALL] OR<br>"colon neoplasm"[ALL] OR<br>"rectal neoplasm"[ALL] OR<br>"colon polyp"[ALL] OR<br>"rectum polyp"[ALL] OR<br>"rectum adenocarcinoma"[ALL] OR<br>"colon adenocarcinoma"[ALL]) AND<br>("vitamin d"[MeSh] OR<br>"vitamin d"[ALL] OR<br>"ergocalciferols"[MeSh] OR<br>"ergocalciferols"[ALL] OR<br>"25-hydroxyvitamin D"[ALL] OR<br>"vitamin D2"[ALL] OR<br>"vitamin D3"[ALL] OR<br>"cholecalciferol"[ALL])'
**COCHRANE SEARCH**
#1 MeSH descriptor: [Colorectal Neoplasms] explode all trees<br>#2 (colorectal neoplasms):ti,ab,kw<br>#3 (colorectal cancer):ti,ab,kw<br>#4 (colon cancer):ti,ab,kw<br>#5 (rectal cancer):ti,ab,kw<br>#6 (colon neoplasm):ti,ab,kw<br>#7 (rectal neoplasm):ti,ab,kw<br>#8 (colon polyp):ti,ab,kw<br>#9 (rectum polyp):ti,ab,kw

#10	(rectum adenocarcinoma):ti,ab,kw
#11	(colon adenocarcinoma):ti,ab,kw
#12	#1 OR #2 OR #3 OR #4 OR #5 OR #6 OR #7 OR #8 OR #9 OR #10 OR #11
#13	MeSH descriptor: [Hydroxycholecalciferols] explode all trees
#14	(vitamin d):ti,ab,kw
#15	(ergocalciferols):ti,ab,kw
#16	(25(OH)D):ti,ab,kw
#17	("25-hydroxyvitamin-D"):ti,ab,kw
#18	(vitamin D2):ti,ab,kw
#19	(vitamin D3):ti,ab,kw
#20	(cholecalciferol):ti,ab,kw
#21	#13 OR #14 OR #15 OR #17 OR #18 OR #19 OR #20
#22	#12 AND #21

Table S2. Influence analysis using the leave-one out approach for the meta-analyses assessing the association between vitamin D intake (highest versus lowest categories) and the risk of colon, rectal and colorectal cancer.

Author	OR/HR (95% CI)	% change OR/HR	I^2	Tau^2
**CCS – CRC – Dietary Vit D – All subjects**	**0.75 (0.67; 0.85)**	**NA**	**46***	**0.0184**
Omitting Levi et al. 2000[¥]	0.73 (0.66; 0.80)	-3.35	17	0.0042
Omitting Jenab et al. 2010	0.75 (0.66; 0.85)	-0.84	49*	0.0209
Omitting Key et al. 2011	0.75 (0.66; 0.86)	-0.47	50*	0.0221
Omitting Sun et al. 2012	0.75 (0.66; 0.86)	-0.39	50*	0.023
Omitting Mizoue et al. 2008	0.75 (0.66; 0.86)	-0.26	50*	0.0217
Omitting Boutron et al. 1996	0.75 (0.66; 0.85)	-0.07	50*	0.0207
Omitting La Vecchia et al. 1997	0.75 (0.66; 0.87)	-0.05	50*	0.0245
Omitting Theodoratou et al. 2008	0.75 (0.66; 0.87)	-0.05	50*	0.0246
Omitting Olsen et al. 1994	0.75 (0.67; 0.85)	-0.03	50*	0.0206
Omitting Ferraroni et al. 1994	0.76 (0.66; 0.87)	0.43	51*	0.0234
Omitting Banqué et al. 2012	0.77 (0.69; 0.86)	2.15	35	0.0112
Omitting Zhang et al. 2020	0.78 (0.70; 0.87)	3.82	23	0.007
**CCS – CC – Dietary Vit D – All subjects**	**0.82 (0.67; 0.98)**	**NA**	**55**	**0.0396**
Omitting Kampman et al. 2000 [Men] [¥]	0.75 (0.65; 0.86)	-8.47	10*	0.0034
Omitting Kampman et al. 2000 [Women]	0.79 (0.65; 0.96)	-3.42	55*	0.0372
Omitting Jenab et al. 2010	0.8 (0.65; 0.98)	-2.39	58*	0.0427
Omitting Ferraroni et al. 1994	0.83 (0.66; 1.04)	1.84	61*	0.0545
Omitting Mizoue et al. 2008	0.83 (0.68; 1.03)	2.40	60*	0.0476
Omitting Lipworth et al. 2009	0.84 (0.68; 1.04)	2.97	60*	0.0491
Omitting Pritchard et al. 1996	0.84 (0.69; 1.03)	3.49	58*	0.0412
Omitting Zhang et al. 2020	0.85 (0.68; 1.05)	3.97	55*	0.0472
**CCS – RC – Dietary Vit D – All subjects**	**0.67 (0.51; 0.87)**	**NA**	**70***	**0.1127**
Omitting Lipworth et al. 2009[¥]	0.62 (0.48; 0.79)	-7.57	59*	0.0699
Omitting Slattery et al. 2004 [Men]	0.63 (0.48; 0.82)	-6.23	66*	0.0953
Omitting Mizoue et al. 2008	0.65 (0.48; 0.88)	-2.39	74*	0.1301
Omitting Ferraroni et al. 1994	0.66 (0.48; 0.90)	-1.52	74*	0.1427
Omitting Jenab et al. 2010	0.67 (0.50; 0.90)	0.79	74*	0.1271
Omitting Slattery et al. 2004 [Women]	0.69 (0.51; 0.92)	2.88	73*	0.1241
Omitting Pritchard et al. 1996	0.69 (0.52; 0.92)	3.06	73*	0.1212
Omitting Hosseinzadeh et al. 2019	0.71 (0.54; 0.93)	5.62	70*	0.1051
Omitting Zhang et al. 2020	0.71 (0.54; 0.93)	6.42	63*	0.092
**PCS – CRC – Dietary Vit D – All subjects**	**0.94 (0.79; 1.11)**	**NA**	**7**	**0.015**
Omitting Ishihara et al. 2008 [Women]	0.89 (0.77; 1.02)	-5.20	14	0.0037
Omitting Järvinen et al. 2001	0.90 (0.78; 1.03)	-4.17	19	0.0051
Omitting Ishihara et al. 2008 [Men]	0.95 (0.78; 1.16)	1.67	50⸸	0.0228
Omitting McCullough et al. 2003	0.97 (0.77; 1.21)	3.15	50⸸	0.0289
Omitting Park et al. 2007 [Men]	0.98 (0.77; 1.23)	4.17	50⸸	0.0321
Omitting Park et al. 2007 [Women]	0.99 (0.83; 1.19)	6.09	23	0.0099
**PCS – CRC – Dietary Vit D – Women**	**0.93 (0.81; 1.07)**	**NA**	**20**	**0.007**
Omitting Terry et al. 2002	0.90 (0.77; 1.05)	-3.67	16	0.0062
Omitting Ishihara et al. 2008	0.90 (0.80; 1.02)	-3.50	0	0
Omitting McCullough et al. 2003	0.92 (0.79; 1.07)	-1.28	27	0.0098
Omitting Lin et al. 2005	0.94 (0.80; 1.1)	0.49	33	0.0125
Omitting Keese et al. 2005	0.95 (0.81; 1.11)	1.33	33	0.0128
Omitting Martínez et al. 1996	0.96 (0.82; 1.13)	3.00	29	0.012
Omitting Park et al. 2007	1.00 (0.86; 1.15)	6.63	0	0

Data show the leave-one out approach for the different meta-analyses with more than 4 study comparisons. Meta-analyses were performed using generic inverse-variance random effects models. Legend for studies:

[¥], reported as an outlier. **Legend for I^2 column:** *, P-value ≤0.05; ⸸, P-value >0.05 and <0.10. **Abbreviations: CC**, colon cancer; **CCS**, case-control studies; **CI**, confidence interval; **CRC**, colorectal cancer; **NA**, not applicable; **PCS**, prospective cohort studies; **RC**, rectal cancer; **Vit**, vitamin.

Figure S1. Forest plot for the association between vitamin D intake (highest versus lowest categories) and risk of colorectal cancer (case-control and prospective studies).

A) Case-control studies – Supplemental vitamin D - All subjects

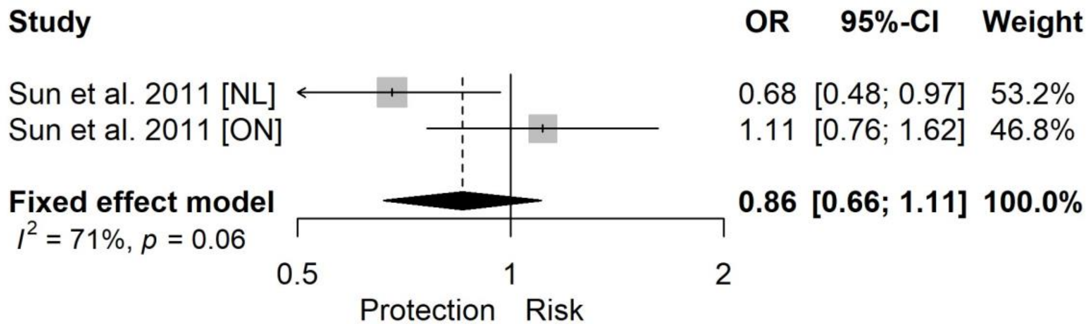

B) Case-control studies - Total vitamin D - All subjects

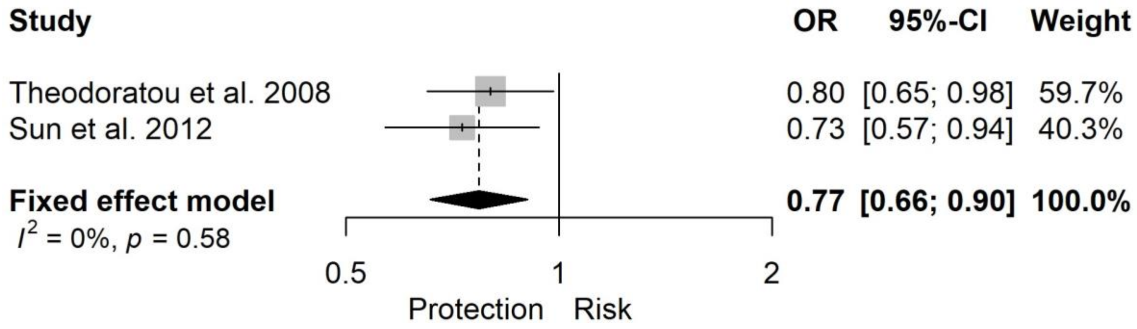

C) Prospective cohort studies - Dietary vitamin D - Men

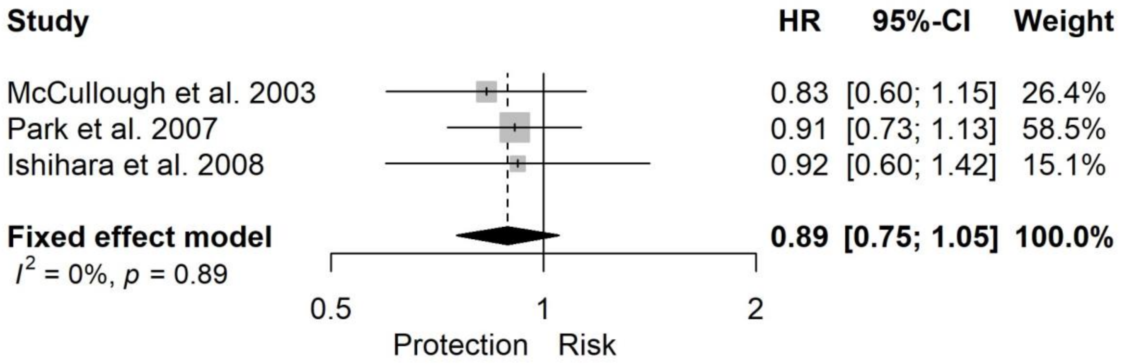

D) Prospective cohort studies - Dietary vitamin D - Women

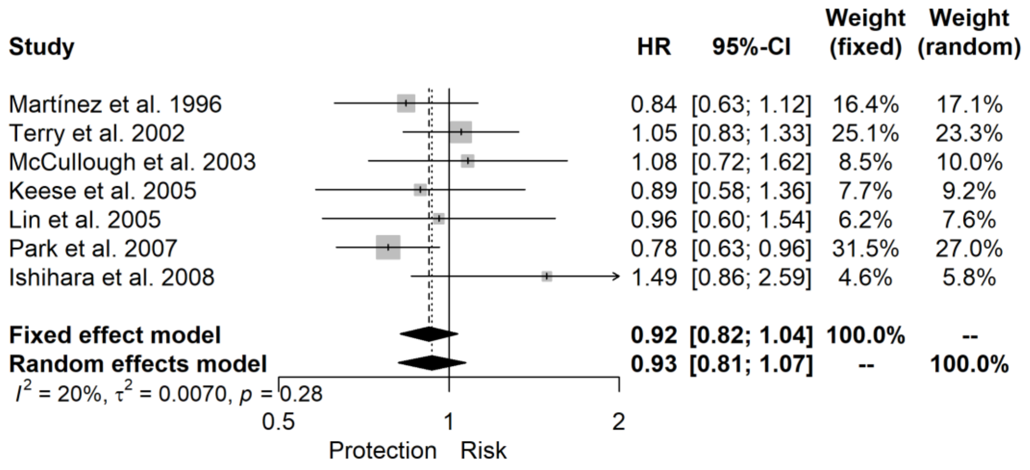

E) Prospective cohort studies - Supplemental vitamin D - All subjects

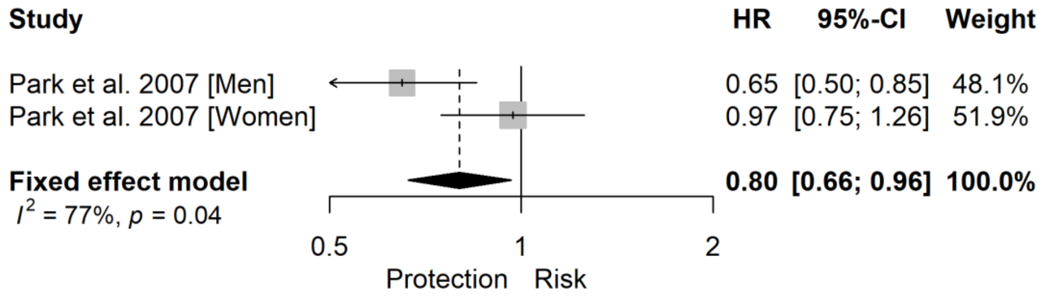

F) Prospective cohort studies - Supplemental vitamin D – Women

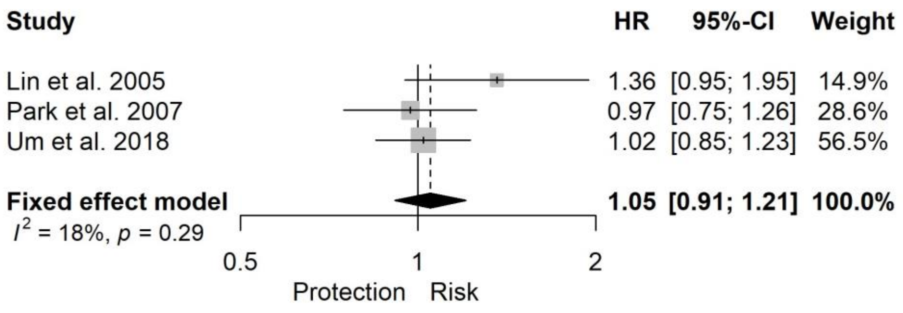

G) Prospective cohort studies - Total vitamin D - All subjects

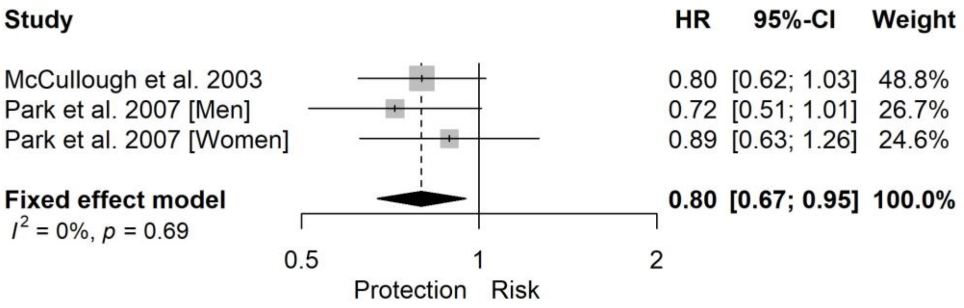

H) Prospective cohort studies - Total vitamin D – Men

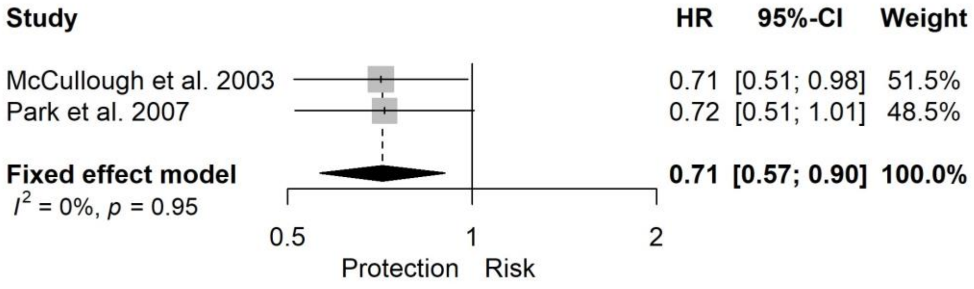

I) Prospective cohort studies - Total vitamin D - Women

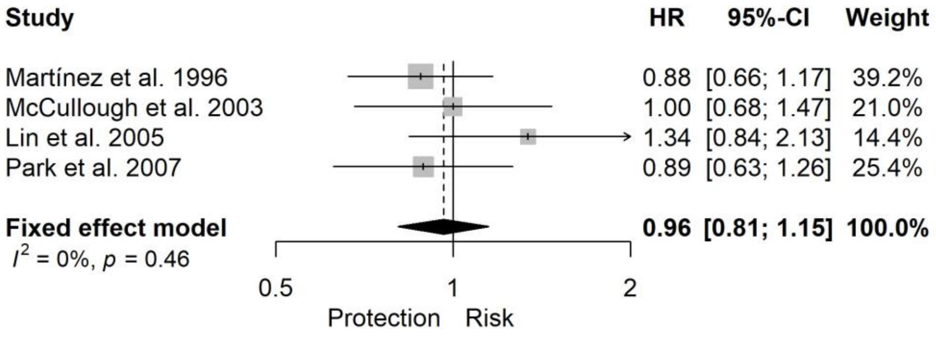

Meta-analyses were constructed using generic inverse-variance fixed-effects model (for meta-analysis with less than 5 studies) or both fixed and random-effects model (for meta-analysis with 5 or more studies). **Abbreviations: CI**, confidence interval; **HR**, hazard ratio; **OR**, odds ratio.

Figure S2. Forest plot for the association between vitamin D intake (highest versus lowest categories) and risk of colon cancer (case-control and prospective studies).

A) Case-control studies – Dietary Vit D - All subjects

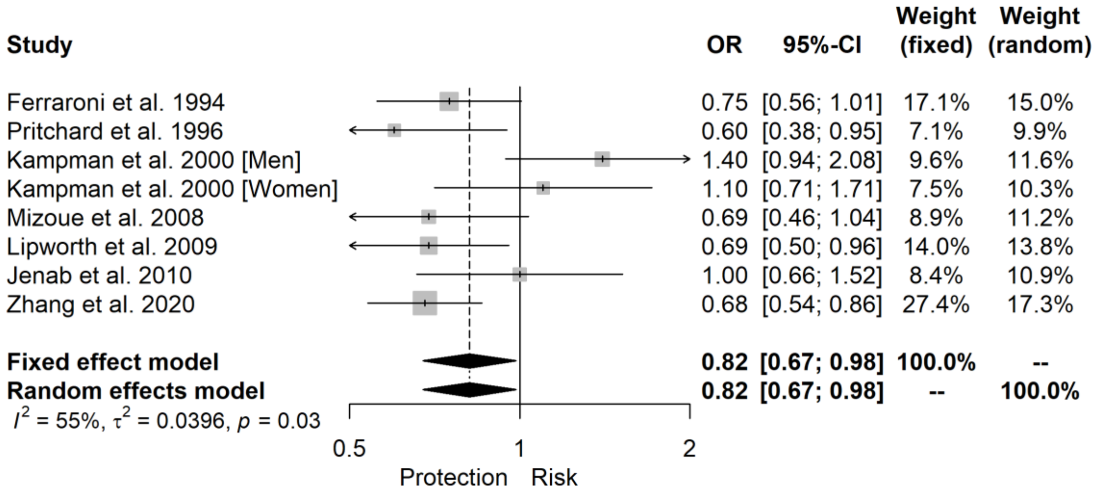

B) Case-control studies - Dietary vitamin D – Men

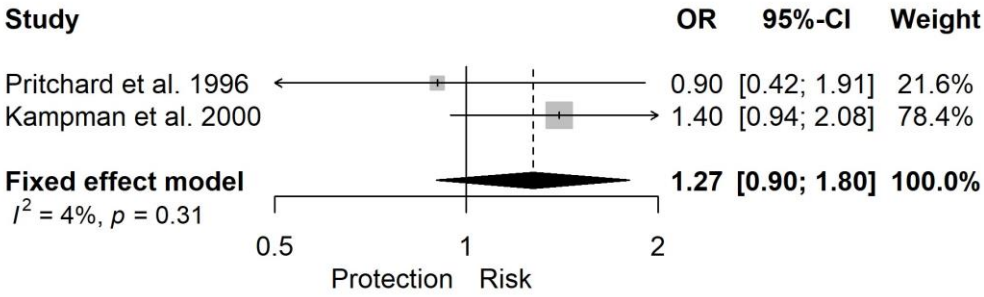

C) Case-control studies - Dietary vitamin D – Women

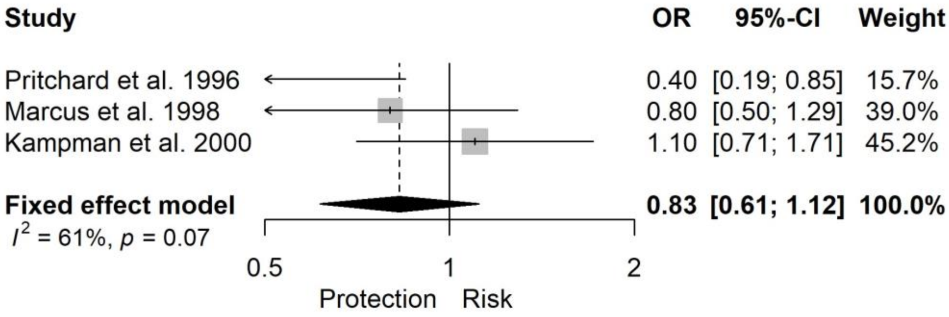

D) Case-control studies - Supplemental vitamin D - All subjects

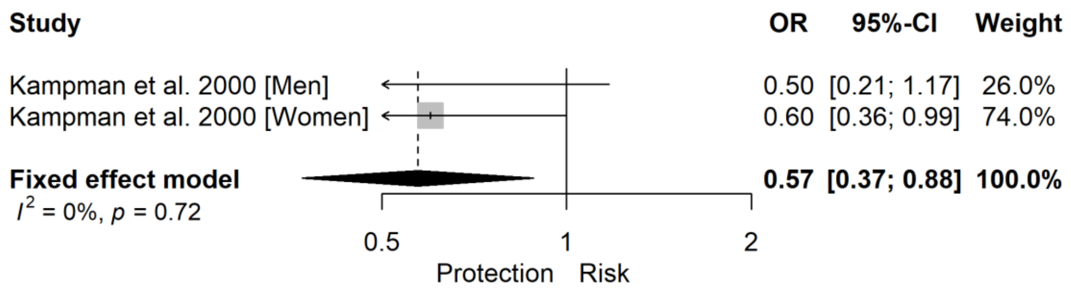

E) Case-control studies - Supplemental vitamin D - Women

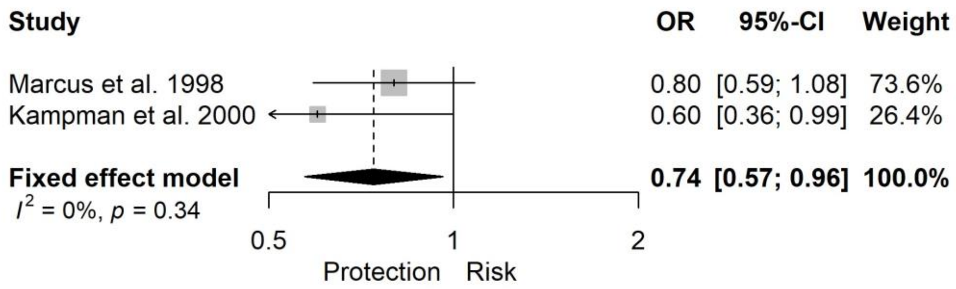

F) Prospective cohort studies - Dietary vitamin D – Women

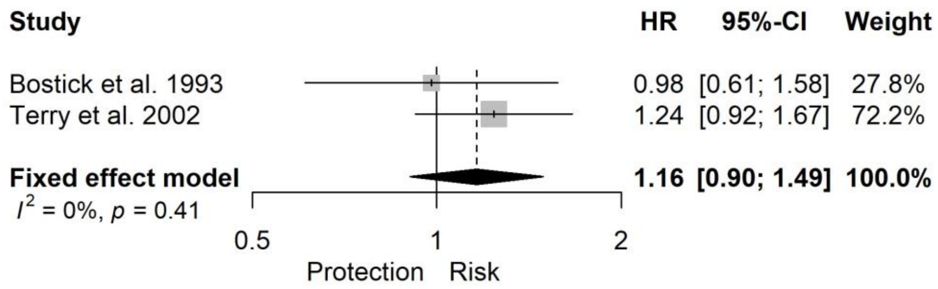

Meta-analyses were constructed using generic inverse-variance fixed-effects model (for meta-analysis with less than 5 studies) or both fixed and random-effects model (for meta-analysis with 5 or more studies). **Abbreviations: CI**, confidence interval; **HR**, hazard ratio; **OR**, odds ratio.

Figure S3. Forest plot for the association between vitamin D intake (highest versus lowest categories) and risk of rectal cancer (case-control studies).

A) Case-control studies - Dietary vitamin D - All subjects

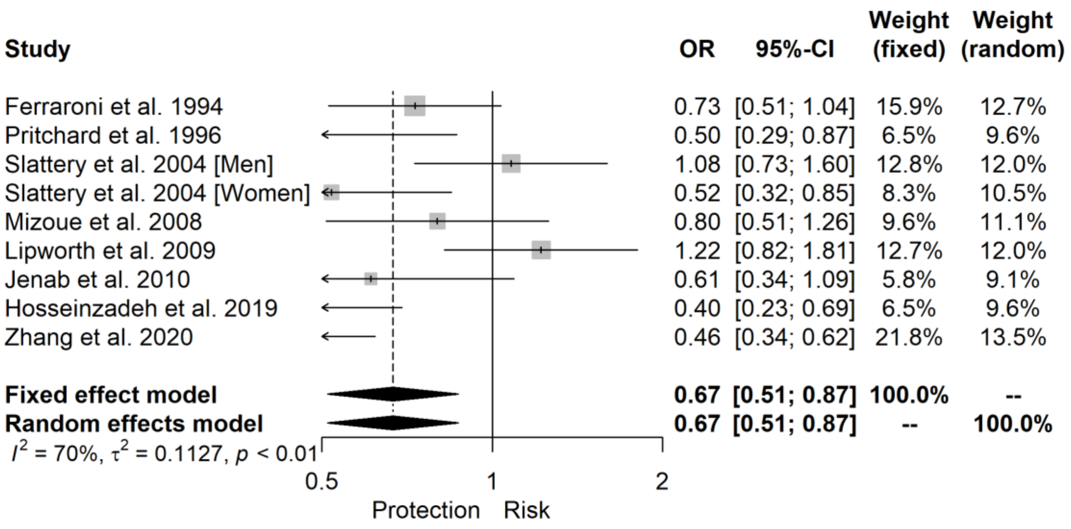

B) Case-control studies - Dietary vitamin D - Men

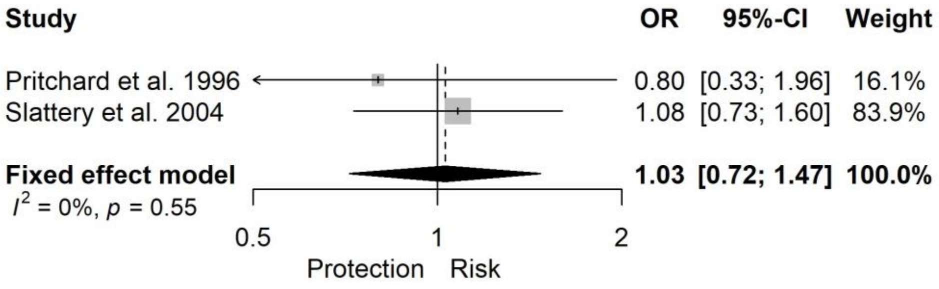

C) Case-control studies - Dietary vitamin D - Women

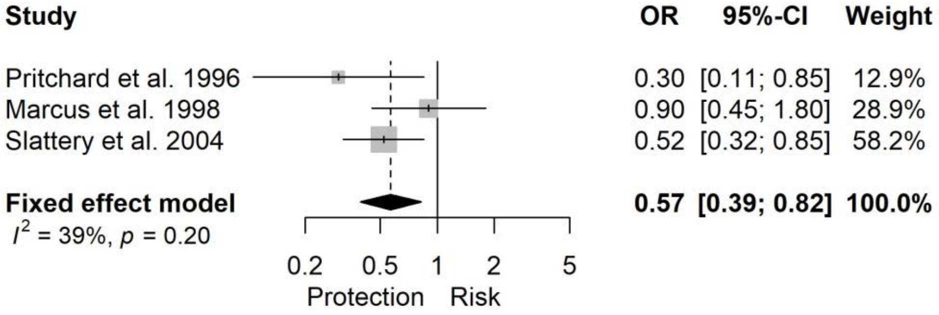

Meta-analyses were constructed using generic inverse-variance fixed-effects model (for meta-analysis with less than 5 studies) or both fixed and random-effects model (for meta-analysis with 5 or more studies). **Abbreviations: CI**, confidence interval; **HR**, hazard ratio; **OR**, odds ratio.

Figure S4. Graphic display of heterogeneity (GOSH) plot analyses for the different meta-analyses

A

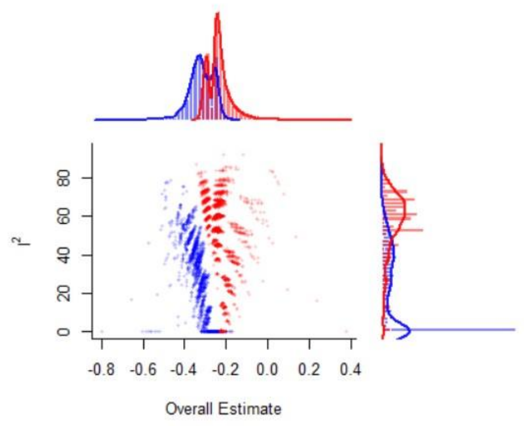

B

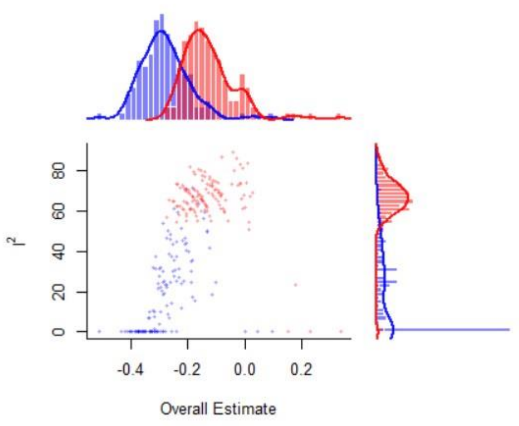

C

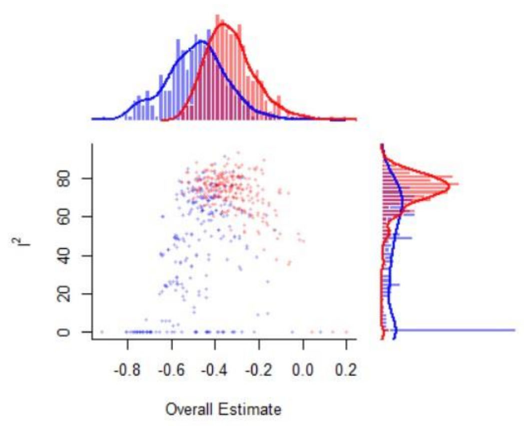

D

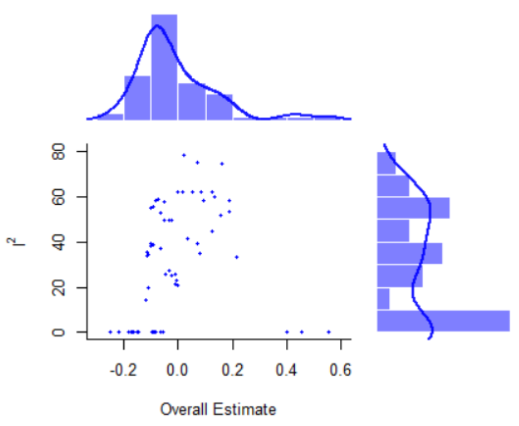

E

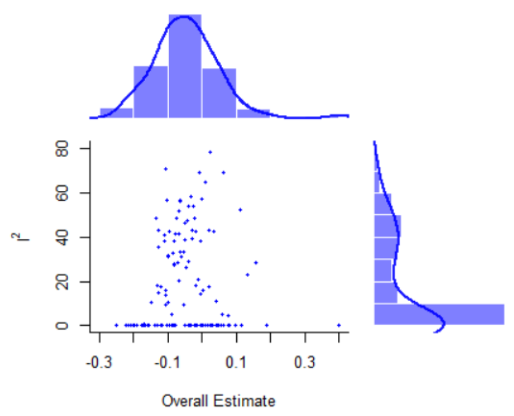

All the iterative meta-analyses ($2^{studies} - 1$ individual analyses) were constructed using generic inverse-variance random-effects model. **Blue** was used for plotting results considering iterative combinations excluding the outliers (Levi et al. 2000 in **A**; Kampman et al. 2000 [Men] in **B**; and Lipworth et al. 2009 in **C**)), whereas **red** color is used to plot results including each respective outlier. Plots are for: **A.** CCS – CRC – Dietary Vit D – All subjects (12 studies, thus including 4095 possible subsets); **B.** CCS – CC – Dietary Vit D – All subjects (8 studies, thus including 255 possible subsets); **C.** CCS – RC – Dietary Vit D – All subjects (9 studies, thus including 511 possible subsets); **D.** PCS – CRC – Dietary Vit D – All subjects (6 studies, thus including 63 possible subsets); **E.** PCS – CRC – Dietary Vit D – Women (7 studies, thus including 127 possible subsets).**Abbreviations: CC**, colon cancer; **CCS**, case-control studies; **CRC**,

colorectal cancer; **GOSH,** graphic display of heterogeneity.**NA**, not applicable; **PCS**, prospective cohort studies; **RC**, rectal cancer; **Vit**, vitamin.

Figure S5. Funnel plot for detecting publication bias in the meta-analysis of case-control studies assessing the association between dietary vitamin D and colorectal cancer in all the subjects

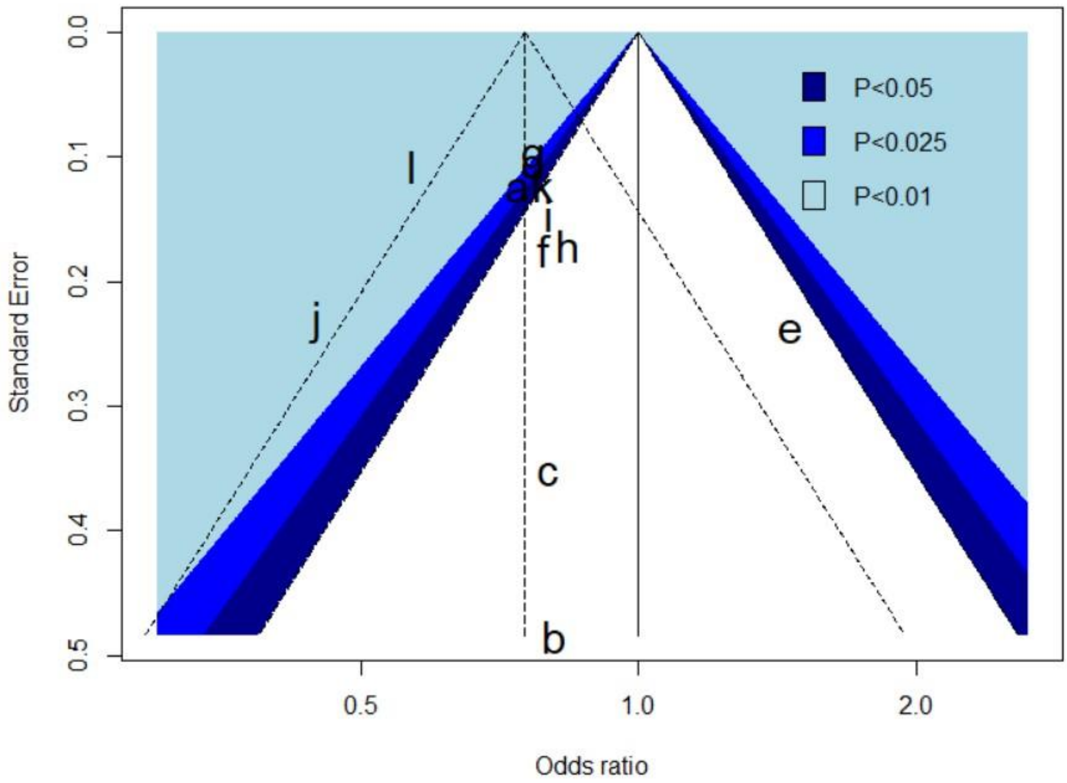

This contour-enhanced funnel plot shows the standard error and odds ratio for each of the studies of the meta-analysis. The different contour colors indicate the significance level (*see* legend in the plot) into which the effects size of each study falls. Egger's test for funnel plot asymmetry was not significant (P=0.508). **Legend:** (**a**) Ferraroni et al. 1994; (**b**) Olsen et al. 1994; (**c**) Boutron et al. 1996; (**d**) La Vecchia et al. 1997; (**e**) Levi et al. 2000; (**f**) Mizoue et al. 2008; (**g**) Theodoratou et al. 2008; (**h**) Jenab et al. 2010; (**i**) Key et al. 2011; (**j**) Banqué et al. 2012; (**k**) Sun et al. 2012; (**l**) Zhang et al. 2020. **Note that studies: a, d, g and k overlap in the plot.**
